# Unhandled horses classified with broken/unbroken test (BUT) exhibit longer avoidance, flight reactions, and displacement behaviors when approached by humans

**DOI:** 10.3389/fvets.2022.1022255

**Published:** 2022-09-26

**Authors:** Maria Giorgia Riva, Lucia Sobrero, Laura Menchetti, Michela Minero, Barbara Padalino, Emanuela Dalla Costa

**Affiliations:** ^1^Department of Veterinary Medicine and Animal Science (DIVAS), University of Milan, Lodi, Italy; ^2^Department of Agricultural and Food Sciences, Alma Mater Studiorum–University of Bologna, Bologna, Italy

**Keywords:** horse, behavior, BUT test, unhandled horse, transport, welfare

## Abstract

Horses with a low level of tameness are at higher risk for transport-related disease and injury; hence, European regulations for the protection of animals during transport (EC 1/2005) are stricter for unhandled (unbroken) horses. However, the regulation does not provide adequate tools for unhandled horse identification. The Broken/Unbroken Test (BUT) was developed and validated to easily identify whether a horse is broken (handled) or not. As a further validation step, the aim of this study was to assess whether there is any correspondence between the BUT classification and the behavioral response of the horse. A total of 100 healthy Italian Heavy Draft horses were video recorded when assessed with the BUT. In total, 90 videos (48 handled and 42 unhandled horses) matched the inclusion criteria and were assessed. The behavior of each horse was evaluated by three observers blinded as to the horses' experience with a focal animal continuous recording method. Behaviors were classified in four categories: stress, avoidance, displacement, and aggression. A Mann–Whitney test was used to identify differences in behavioral patterns between horses classified as handled or unhandled with the BUT. Unhandled horses showed not only a significantly longer time to be approached by the handler but also more avoidance and flight reactions (*p* < 0.001). Unhandled horses showed significantly longer displacement behaviors, such as sniffing (*p* < 0.001). These findings further validate the BUT classification and confirm that horses classified as unhandled are more prone to show avoidance and flight reactions when approached by humans. For this reason, the adoption of the BUT could be helpful to minimize humans' horse-related injuries and, if applied regularly before loading, to contribute to safeguard the welfare of horses during transport.

## Introduction

Transportation is a stressful event for horses (1–3). Because of their ethological characteristics, horses with a low level of tameness are at a higher risk for transport-related disease and injury (4). Regulation EC 1/2005 recognizes that broken and unbroken horses have different needs and adaptability skills. Therefore, it provides stricter rules for the transportation of unbroken animals and defines them as horses that “cannot be tied or led by a halter without causing avoidable excitement, pain, and suffering.” However, the lawgiver does not provide any tools for this classification, and, consequently, official authorities can find it difficult to verify regulatory compliance. Menchetti et al. (5) developed and validated a reliable behavioral test to identify whether a horse is broken or not, the Broken/Unbroken Test (BUT). The term “broken” (and its contrary, “unbroken”) was used in the study of Menchetti et al. (5) to be consistent with the terminology used in Regulation EC 1/2005 to describe horses with a basic level of handling and training, familiar with humans' contact. For this reason, the words “broken” and “handled” were used as synonyms without any reference to the fact that these horses were or were not trained for riding or driving that is irrelevant for the specific purposes. The BUT consists of two phases: approaching and haltering, and leading the horse. Each part should be scored by an observer. In the original study, the BUT was validated by comparing the score given by observers, who were blinded as to the horses' experience, to a gold standard, the “expert judgment” (the judgment of the senior veterinarian who performed all the tests), and to physiological parameters (heart and respiratory rate, eye temperature). The BUT score showed excellent interobserver, intraobserver, and test-retest reliability, but these findings were preliminary, and the test needs further validation. In the original validation of the BUT (5), the only behavioral evaluation that had been considered was the measurement of the “avoidance distance” (i.e., the distance between tester and horse) during the approaching and haltering phase. Unhandled horses tend to show a strong herd instinct, as reported by Knowles et al. (4). In their study on unbroken ponies' transport, they reported that individual horses were difficult to separate from the group and tended to keep a certain distance between them and the handler, attempting to kick or to bite if they felt confined. Therefore, although time-consuming, the evaluation of behavior can be used as an effective method to identify unhandled horses and to validate the BUT test. Behavior assessment can also help in understanding whether the horse perceives the interaction with humans in a positive or negative way, providing a more accurate and comprehensive assessment of stress responses (6–8). As it has been already demonstrated for companion (9–11) and farm animals (12–17), early experience with humans has an impact on the way these animals behave and relate with humans or even reduces fear responses toward them. Moreover, horse–human relationship depends, among others, also on horses' training and working conditions (19) and, even if they do discriminate against familiar figures, experience with humans tends to be generalized from familiar to unfamiliar ones (18, 19). Behavior is also considered the most immediate indicator of animal welfare in several species (20), including horses (21). Unbroken horses have a different capacity to cope with challenging situations; they tend to react with a higher level of fear, excitability, and arousal activation and develop negative emotional states and distress responses (19, 22). This is also particularly relevant for human safety since fear reactions are the leading cause of horse-related injuries (23, 24). As shown by Sankey et al.' study (19), trained horses have an enhanced acceptance toward an approaching human, and they tend to approach humans more rapidly when compared to naïve subjects. The aim of this study was to assess whether horses classified as handled or unhandled with the BUT test showed significantly different behavior. Our hypothesis was that handled animals would show less stress, avoidance, displacement, and aggressive behaviors than unhandled ones during the BUT.

## Materials and methods

### Video recordings and BUT classification

A total of 100 healthy Italian Heavy Draft horses (16 males and 84 females, including 14 broodmares with foals, aged 1–25 years) were assessed with the Broken/Unbroken Test (BUT). These assessed horses were either born on the farm or had been kept on it for at least 3 months before the assessment. They had been reared for various purposes (breeding, meat production, family enjoyment, and showing). No particular behavioral problems (i.e., aggression and stereotypy) have been reported by the owners. The required sample size to validate the BUT test was calculated using the ICC procedures, assuming a 95% CI of width Wk = 0.15, a planning value of ICC (ICC plan) = 0.7 and k = 4 raters. Demographic data (i.e., age and sex) and level of handling of the horses were provided by the owners. More details are given in the study by Menchetti et al. (5). The BUT consisted of attributing a score to the horse during two phases: the approach and haltering test (AHT), and the handling test (HT); all these phases were performed by the same tester, a veterinarian with more than 20 years of experience in horse behavior who was trained for the procedure. The tester was blinded as to the horses' experience. For each horse, the maximum time allowed for completing the BUT was 10 min (5 min for each phase) and they were all tested in a familiar environment, the paddock/pen where they were usually kept (test area). The tester entered the test area and walked toward the horse slowly with the halter in her hand, approaching and trying to halter the horse (AHT); if it was possible to halter the horse within the maximum time, the tester started the second phase of the test (HT); otherwise, the test ended. During HT, the tester tried to lead the horse three steps forward and three steps backward; for further details, see Menchetti et al. (5). Videos were recorded by Menchetti and et al. (5) using a digital video camera recorder (HDR-CX115E, Sony, China). From a total of 165 videos (65 horses were re-tested with a 3-week interval), 90 videos were selected as they matched the following criteria: in the first BUT evaluation, the assessed horse was clearly visible for the entire duration of the test. Videos were renamed and coded using casual numbers by an author not involved in the study. Finally, selected videos were divided into two groups based on the BUT classification: handled (*N* = 48) and unhandled (*N* = 42).

#### Behavioral evaluation

Three observers (two veterinarians specializing in ethology and animal welfare, and a fourth-year veterinary student with experience in horse behavior), blind to the experimental design and group allocation, analyzed the videos. They previously attended a specific training that ended only when they reached an optimal interobserver agreement. Horse behavior was evaluated with a focal animal continuous recording method (25), using the software Solomon Coder (beta 12.09.2004, copyright 2006–2008 by András Péter). The ethogram used for behavior analysis was adapted from McGreevy and McLean (26), Corgan et al. (27), and Young et al. (28). To better define some specific stress behaviors, a few more published studies were used (27, 29, 30). The resulting ethogram is reported in Table 1. Depending on the behavior, they were distinguished into “event” or “state”; then evaluated for frequency or duration. Behaviors were grouped in four categories: stress (defecation, urination, head tossing, tail swishing), avoidance (avoid side, back, front, and flight reaction), displacement (irrelevant to the behavioral context: eating, sniffing, licking/chewing, interaction with other horses) and aggressive (kicking or attempting to kick, biting or attempting bite, pushing).

**Table 1:** Ethogram for the evaluation of horse behavior during the broken/unbroken test.

**Behavior**	State/Event^*^	**Definition**	**Category**
Avoid side	S	Avoiding the tester by evasive steps to the side	Avoidance behavior
Avoid back	S	Avoiding the tester by evasive steps back-wards, backing up	Avoidance behavior
Avoid front	S	Avoiding the tester by evasive steps on-wards	Avoidance behavior
Avoid flight	S	Sudden movement, feet moving faster a walk/trot away	Avoidance behavior
Turning the head away	E	The horse turns his head and neck to the right or to the left appearing to look away avoiding the tester	Avoidance behavior
Eating	S	The horse eats hay or grass, not paying attention to the tester	Displacement behavior
Sniffing	S	The horse sniffs around, it sniffs the halter, the rope, or the tester	Displacement behavior
Interaction with other horses	S	The horse interacts with one of the other horses of the herd, they sniff (or groom) each other	Displacement behavior
Licking/Chewing	E	The horse opens the mouth with extension and retraction of tongue, lip smacking without tongue extension, lateral jaw movements involving partial opening of the lips (6)	Displacement behavior
Defecation	E	The horse defecates	Stress behavior
Urination	E	The horse urinates	Stress behavior
Head tossing	E	The horse shakes its head suddenly, violently, and frequently	Stress behavior
Tail wishing	E	The horse wishes his tail rapidly	Stress behavior
Kicking or attempt kick	E	The horse put his back legs toward the tester, and tries to kick or kick (one or both hind legs lift off the ground and rapidly extend backwards, the forelegs support the weight of the body, and the neck is often lowered)	Aggressive behavior
Biting or attempt bite	E	The horse puts the ears flat back, and bites or tries to bite the tester	Aggressive behavior
Pushing	E	The horse pushes with this head, neck or shoulder the tester away	Aggressive behavior

* S, state; E, event.

### Statistical analysis

Behavioral data were analyzed using SPSS 27 (SPSS Inc., Chicago, USA). Behaviors recorded as states were analyzed as duration (in seconds), while those recorded as events were examined as frequency of occurrence. Then, descriptive statistics (mean and standard error) were calculated; then behavioral data was tested for normality and homogeneity of variance using the Kolmogorov–Smirnov and Levene test, respectively. Data were not normally distributed and therefore a Mann–Whitney test was used to identify differences in behavioral patterns between horses classified as handled and unhandled with the BUT. The power achieved by these tests (calculated using G^*^Power, version 3.1), setting a one-tailed test, medium effect size, and α = 5, was 87%. Differences were considered statistically significant if *p* < 0.05.

## Results

**Figure 1:**
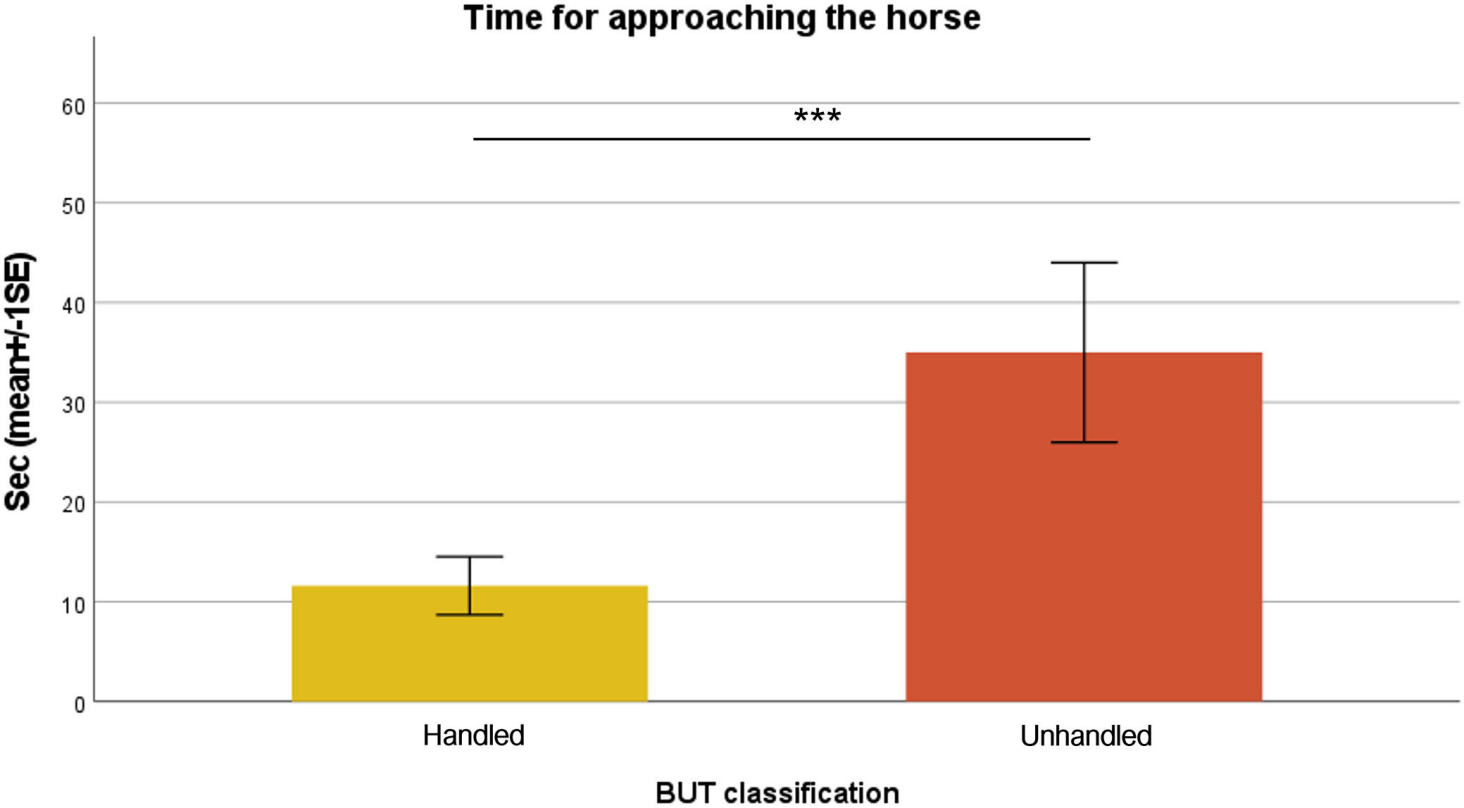
Mean and standard errors of time for approaching(s). The level of significance was set at ^***^p < 0.001.

**Figure 2:**
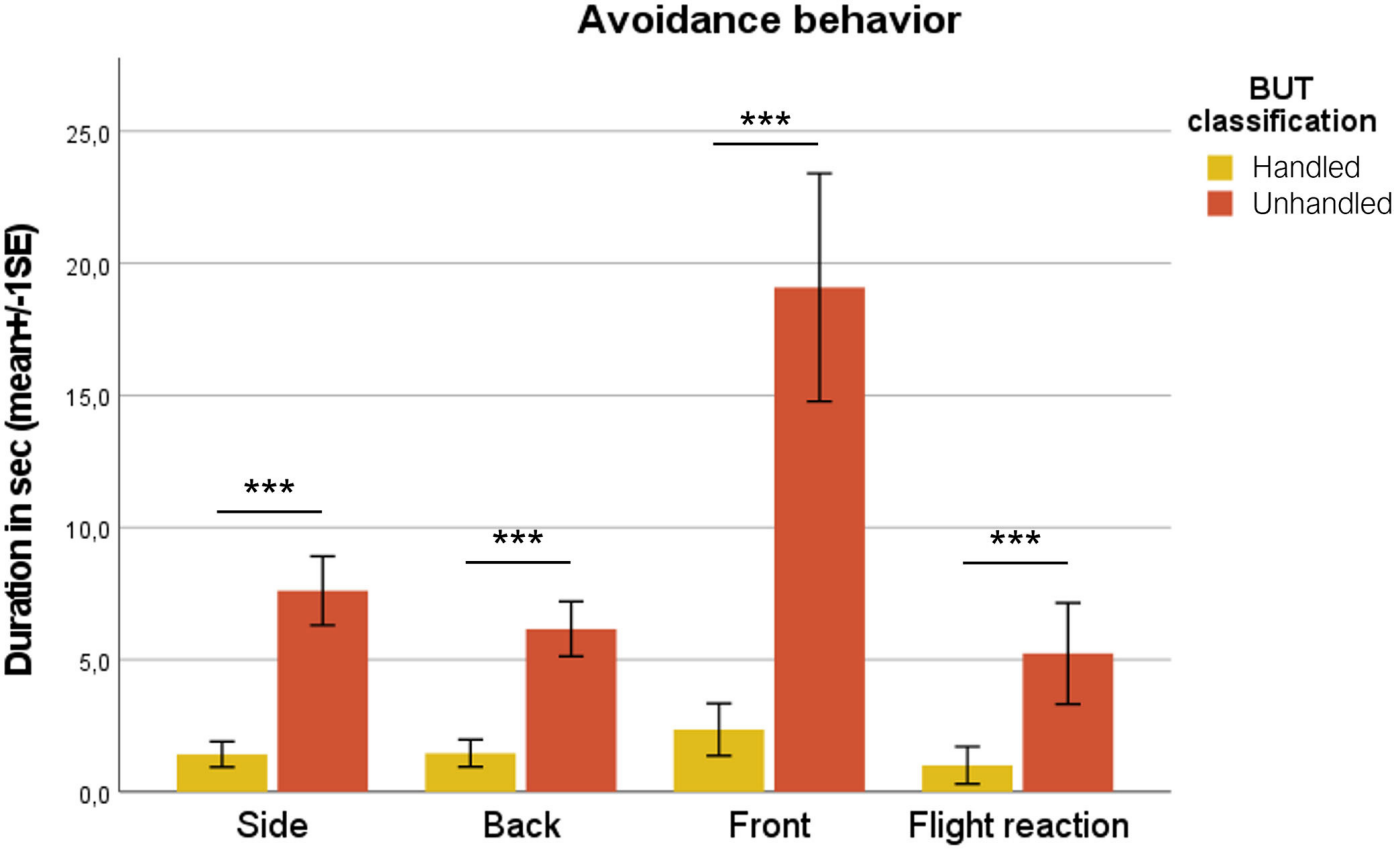
Mean duration in seconds (1 SE) referred to avoidance behaviors in broken and unbroken group. Mann–Whitney test; ^***^p < 0.01.

**Figure 3:**
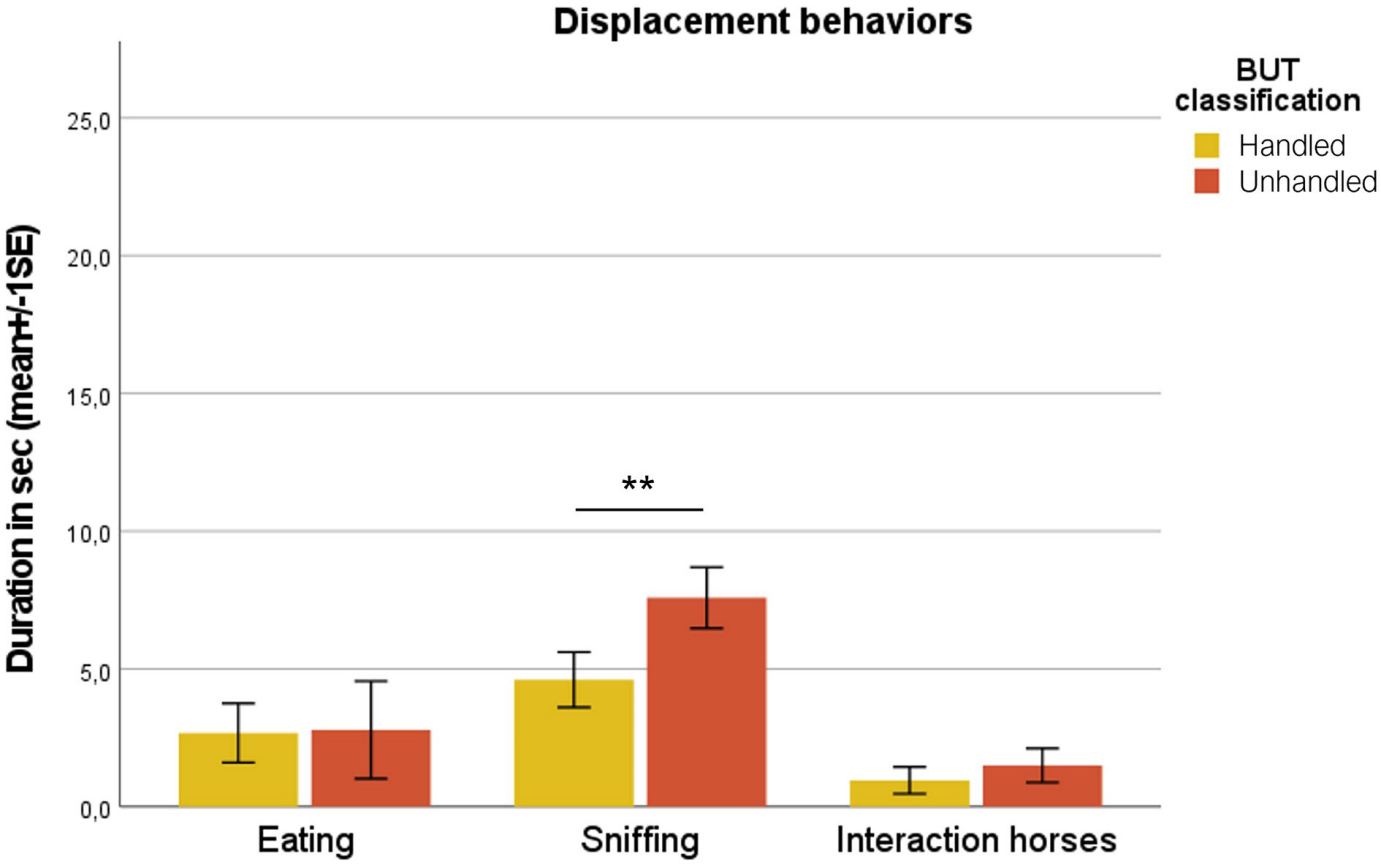
Mean durations in seconds (1 SE) referred to displacement behaviors in broken and unbroken group. Mann–Whitney test; ^**^p < 0.001.

**Figure 4:**
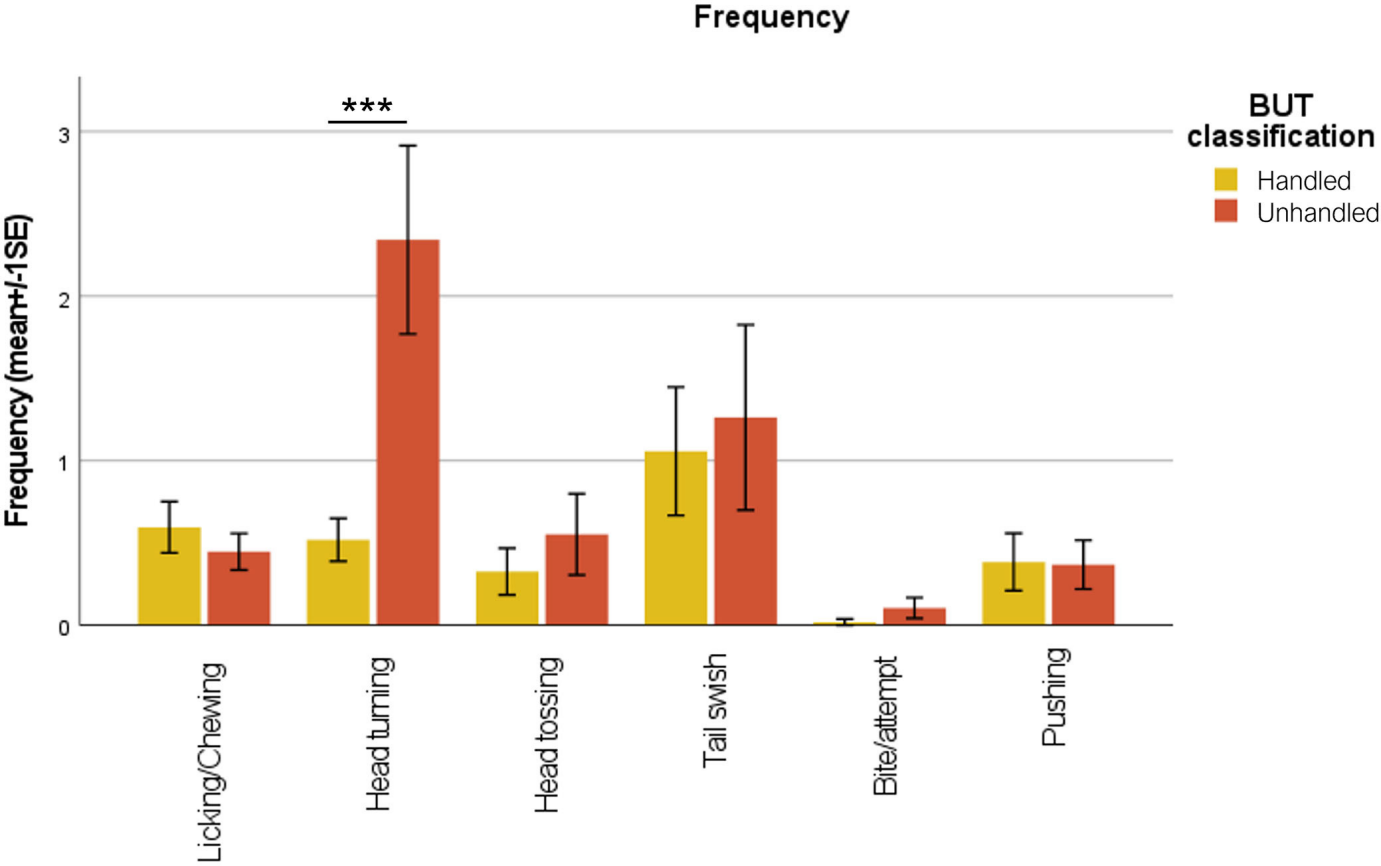
Mean frequencies referred to behaviors recorded as events. Mann–Whitney test; ***p < 0.001.

Unhandled horses showed a significantly longer time to be approached by the handler (35.00 ± 9.01 s), compared to handled ones (11.62 ± 2.91 s; Mann–Whitney test; *p* < 0.001) (Figure 1). Considering behavioral categories, mean durations in seconds (± SE) of avoidance behaviors (avoid side, back, front, and flight reaction) are reported in Figure 2. Avoid front was exhibited significantly longer in unhandled horses compared to handled ones, 19.09 ± 4.31 and 2.36 ± 0.99 s, respectively (Mann–Whitney test; *p* < 0.001). Also avoid side and avoid back were shown for a significantly longer duration by unhandled group, 7.61 ± 1.30 and 6.17 ± 1.04 s, respectively (Mann–Whitney test; *p* < 0.001). Compared to the handled group (1.01 ± 0.71 s), unhandled horses showed significantly longer flight reactions (5.24 ± 1.92 s; Mann–Whitney test; *p* < 0.001). Similar results emerged from the analysis of displacement behaviors even if with less value discrepancy between the groups (Figure 3). Sniffing was significantly longer in unhandled horses (7.59 ± 1.11 s) compared to handled horses (4.61 ± 1.16 s; Mann–Whitney test; *p* < 0.01). No differences between groups were found in interaction with other horses (unhandled 1.50 ± 0.62 s; handled 0.96 ± 0.49 s) and eating (unhandled 2.79 ± 1.77 s; handled 2.68 ± 1.08 s). No significant differences between groups were found in the frequencies of licking/chewing, head tossing, tail swishing, bite attempts, and pushing (Figure 4). No horse urinated during the BUT evaluation and only one defecated. Head turning was shown more frequently by unhandled horses compared to handled ones (Mann–Whitney test; *p* < 0.001), 2.34 ± 0.57 and 0.52 ± 0.13 events, respectively.

## Discussion

The aim of this study was to assess whether there is any correspondence between the BUT classification and the behavioral response of the horse when approached by an unknown human to further validate the BUT. The results confirmed our initial hypothesis: horses classified as unhandled with the BUT not only needed a significantly longer time to be approached by humans but also longer or more frequent avoidance (e.g., avoid side, back, front, head turning, and flight reactions) and displacement behaviors (e.g., sniffing). Being prey animals, horses may be remarkably stressed by human forced approaches (31, 32), in particular if these approaches take place in an unfamiliar environment (33). For the purpose of our research, all the horses were tested in their home paddock/field. However, if the test was performed in an unfamiliar environment, for example, at the assembly center, the behavioral responses would probably be more evident: as reported by Zappaterra et al. (34), unhandled horses unloaded at the slaughterhouse tend to show more unpredictable and strong reactions. These results are in accordance with what has been reported by Waiblinger et al. (35) in farmed species: depending on their previous experience, animals naturally tend to perceive human presence as a positive, neutral, or negative stimulus; and, consequently, if not used to human contact, horses may interpret human approach as a potential danger. Knowels et al. (4) also reported that unbroken ponies tend to keep a certain distance from the handler or even attempt to kick or bite if they feel confined, thus justifying the need for longer approaching time. Compared to the handled group, unhandled horses showed more avoidance behaviors (avoid side, back, front, and flight and head turning). Horses are generally considered to be unpredictable, fearful, and flight-wired animals (36). In addition, if they are not used to be approached by humans, they can experience even stronger negative emotional states and a massive activation of the sympathetic nervous system that results in a flight response or other avoidance behaviors (33, 37). In general, unhandled horses tend to react with higher levels of fear and excitability in challenging situations (19, 22). These behavioral traits, combined with horses' size and strength, are particularly relevant for both human and animal safety, being the first cause of accidents involving people (23, 25, 36). Therefore, the correct classification of horses' level of tameness should be considered a fundamental part of good transport practice/procedures both because of protection of animal welfare and because of operators' safety. Training, including self-loading with positive reinforcement, seems to be useful to reduce loading problem. However, even in sport horses, only a minority of owners adopt habituation and self-loading techniques (38, 39) to train horses to be transported. Our results are partly compatible with previous studies (19, 31, 33, 40, 41) reporting that an absent or even sub-optimal human-horse relationship may result both in extended approaching times and in more frequent avoidance behaviors compared to what happens with animals managed to enhance human–animal relationship. On the contrary, these same studies (19, 31, 33, 40, 41) reported a higher frequency of aggressive behaviors, while we did not find any significant difference between the two groups for kicking, biting, and pushing behaviors. This was probably because our tests were conducted in open spaces and, thus, the horses had the possibility to fly away, but also because, as part of the experimental design, the test was stopped at the first sign of pain or if the horse showed a high level of distress or sign of aggression. Finally, among displacement behaviors, sniffing occurred with significantly longer durations in unhandled horses compared to the handled ones. Displacement activities are defined as “movements or actions, which occur outside the behavioral context for which they are originally developed. They appear in conflict situations, and they are generally “unsuccessful” in the sense that they do not serve the biological purpose to which they were originally adapted” (42). Horses are extremely curious animals, in which exploratory behaviors are very developed, especially in foals (42). Despite that, exploring behaviors, like licking, sniffing, or touching with muzzle or tongue parts of the environment, may also be an indication of medium levels of stress, as reported in Young and colleagues' study (28). This finding was already reported in other species (e.g., non-human primates and human subjects), where displacement behaviors seem to be functional to cope with stressful situations (43). Even Root-Bernstein suggested that they could have a functional role in at least some proportion of motivational transitions, for example, high-stress motivational transitions in honeybees (44), as also hypothesized by Wilts in *Gasterosteus aculeatus* (45–47). In the same way, unhandled horses may have expressed this behavior to cope with a novel and challenging situation: the approaching human during the BUT test. Even if BUT reliability (inter- and intra-observer, test-retest reliability) was good as reported in the previous study (5), further investigations were suggested to further validate the test. For example, the environmental context and the breed could influence the behavioral responses and thus compromise the validity of the BUT. In this study, the BUT was applied only to draft horses, which are typically calmer and less reactive compared to other breeds (e.g., Arabian and Thoroughbred horses), and, as previously mentioned, they were all tested in their paddock. Recently, (34) have applied the BUT to draft horses on arrival at a slaughterhouse, supporting the validity of the BUT even in an unfamiliar environment. Further studies are underway to confirm these results, applying the BUT to a larger and more diverse population of horses, namely, dolichomorphic type, housed both in a familiar and unfamiliar environment. Emotion is a subjective experience whose measurement requires behavioral observations, as it cannot be directly assessed (48), and this awareness highlighted the need for an accurate behavioral assessment to get an external validation of our test. In this study, behavioral evaluation confirmed that unhandled horses were more fearful when approached by an unknown human, further validating the BUT as a reliable test to identify broken and unbroken horses. BUT could be included in future legislation as a tool to classify horses to adapt travel conditions to their broken/unbroken status. Moreover, the terms broken/unbroken are not generally accepted; they were replaced by the word “gentling”, which refers to safely and ethically train the horse; they could possibly be replaced by handled and unhandled.

## Conclusion

The definition of “unbroken horse” provided in Reg. 1/2005 on animal transport is susceptible to misinterpretation as no adequate means to make a distinction between handled and unhandled horses are reported.

The BUT could be considered as a valid tool to implement the transport legislation in horses as, in addition to good feasibility and test-retest reliability presents a fair agreement with the behavior shown by horses. Our results confirmed that BUT could guide stakeholders to correctly identify unhandled horses during loading procedures, thus mitigating the human safety and animal welfare risks during transportation and related procedures.

## Data availability statement

The raw data supporting the conclusions of this article will be made available by the authors, without undue reservation.

## Ethics statement

The animal study was reviewed and approved by Committee on Animal Welfare and Ethics of the University of Bologna (Approval number 0001578, 4th of March 2021). Written informed consent for participation was not obtained from the owners because no personal details of the participants were recorded. Verbal informed consent was gained from the breeders prior to taking part in the research.

## Author contributions

MGR, LS, and EDC wrote the article. EDC, LM, MM, and BP reviewed the article. Statistical analysis made by EDC. BP conceived the experimental design. LM and BP collected the data. Videos were analyzed by MGR and LS. All authors contributed to the article and approved the submitted version.

## Funding

This study was funded by Animals' Angels, World Horse Welfare, and Morris Animal Foundation (D21EQ-522).

## Conflict of interest

The authors declare that the research was conducted in the absence of any commercial or financial relationships that could be construed as a potential conflict of interest.

## Publisher's note

All claims expressed in this article are solely those of the authors and do not necessarily represent those of their affiliated organizations, or those of the publisher, the editors and the reviewers. Any product that may be evaluated in this article, or claim that may be made by its manufacturer, is not guaranteed or endorsed by the publisher.
